# Integrated metabolome and transcriptome analyses provide insight into the effect of red and blue LEDs on the quality of sweet potato leaves

**DOI:** 10.3389/fpls.2023.1181680

**Published:** 2023-05-30

**Authors:** Shehu A. Tadda, Chengyue Li, Jintao Ding, Jian’an Li, Jingjing Wang, Huaxing Huang, Quan Fan, Lifang Chen, Pengfei He, John K. Ahiakpa, Benjamin Karikari, Xuanyang Chen, Dongliang Qiu

**Affiliations:** ^1^ College of Horticulture, Fujian Agriculture and Forestry University, Fuzhou, China; ^2^ Department of Agronomy, Federal University Dutsin-Ma, Katsina, Nigeria; ^3^ Agriculture Research Group, Organization of African Academic Doctors (OAAD), Nairobi, Kenya; ^4^ Department of Agricultural Biotechnology, University for Development Studies, Tamale, Ghana; ^5^ College of Agriculture, Fujian Agriculture, and Forestry University, Fuzhou, China; ^6^ Key Laboratory of Crop Biotechnology, Fujian Agriculture and Forestry University, Fujian Province Universities, Fuzhou, China

**Keywords:** leafy vegetable, illumination, metabolome profiling, nutrition, anthocyanin

## Abstract

Red and blue light-emitting diodes (LEDs) affect the quality of sweet potato leaves and their nutritional profile. Vines cultivated under blue LEDs had higher soluble protein contents, total phenolic compounds, flavonoids, and total antioxidant activity. Conversely, chlorophyll, soluble sugar, protein, and vitamin C contents were higher in leaves grown under red LEDs. Red and blue light increased the accumulation of 77 and 18 metabolites, respectively. Alpha-linoleic and linolenic acid metabolism were the most significantly enriched pathways based on Kyoto Encyclopedia of Genes and Genomes (KEGG) pathway analyses. A total of 615 genes were differentially expressed between sweet potato leaves exposed to red and blue LEDs. Among these, 510 differentially expressed genes were upregulated in leaves grown under blue light compared with those grown under red light, while the remaining 105 genes were expressed at higher levels in the latter than in the former. Among the KEGG enrichment pathways, blue light significantly induced anthocyanin and carotenoid biosynthesis structural genes. This study provides a scientific reference basis for using light to alter metabolites to improve the quality of edible sweet potato leaves.

## 1 Introduction

Sweet potato (*Ipomoea batatas* (L.) Lam) is a crop mainly cultivated for its enlarged edible roots and stems. Sweet potato shoots, conversely, have been neglected or restricted to animal feed and are rarely used as vegetables (Cui et al., 2011). Sweet potato leaves have been demonstrated to be more nutritious than their stems, petioles, tubers, and other vegetables (Wang et al., 2016a). Sweet potato is grown all year in China and other tropical regions, which allows for multiple leaf harvests in a single growing season, giving it an advantage over other vegetables (Li et al., 2017). The crop could tolerate extreme weather conditions, which makes it an interesting crop for combating food insecurity (Motsa et al., 2015; Iese et al., 2018). Due to its tenderness, lack of pubescence, and excellent eating quality, the young leaves of recently introduced leafy sweet potato cultivars are in higher demand than leaves from the common tuberous cultivars (Tang et al., 2020). To produce high-quality sweet potato shoots all year, an alternative production system to field conditions is needed.

Light quality and intensity are critical environmental factors affecting photomorphogenesis, plant development, and metabolism. In addition, the ability of plant photosynthetic systems and metabolism to adapt to light fluctuation correlates with their reproductive success and survival rate (Chen et al., 2021). Studies have shown that an increase in light intensity (100-250 µmol m^-2^ s^-1^) enhance the accumulation of chlorophyll and β-carotene in *Dunaliella salina* (Lamers et al., 2010). Red light increases the phenolic and anthocyanin contents of lettuce leaves and pea seedlings (Wu et al., 2007; Li and Kubota, 2009) and the anthocyanin content of basil (Nadeem et al., 2019). Red and green basil exhibited differential responses to blue and red light intensities, where a higher proportion of blue light significantly increased the phytochemical content of the green cultivar (Lobiuc et al., 2017).

Approximately 200,000 metabolites can be produced in several plant cultivars, ecotypes, and species (Lisec et al., 2006; Kusano et al., 2011; Wang et al., 2019). These metabolites are usually measured *via* mass spectrometry (MS) analyses, which usually yield sensitive high-throughput data. MS analysis has been integrated with other analytical systems, including liquid chromatography (LC), capillary electrophoresis (CE), and gas chromatography (GC), to measure various metabolites (Gowda and Djukovic, 2014). In addition, the liquid chromatography−mass spectrometry (LC−MS) metabolomics separation technique has been employed in measuring secondary metabolites in foods and plants (Ma et al., 2013). Other separation techniques employed in metabolomic studies of plants exposed to environmental stress provide high-quality metabolomics data. As such, metabolome profiling is a promising analytical method for evaluating agricultural products, providing a variety of information on various metabolites. Comprehensive metabolome profiling reveals quantitative changes in the metabolite profiles of plants grown under certain environmental conditions (Ghatak et al., 2018).

As a protective mechanism, plants respond to light by regulating genes involved in energy and metabolism. As an important field of biology, transcriptome studies enable scientists to study gene expression at the cellular level to assess transcript-level changes with high precision (Chen et al., 2021). Transcriptome profiling has been used to investigate the effect of blue and red light on the development and growth of several plants, including Norway spruce seedlings (Ouyang et al., 2015), potato (Chen et al., 2021), and synthesis of various metabolites in plants in response to light stress (Xie et al., 2020). Previous studies have focused on understanding genetic, nutritional, metabolic, and transcriptional regulation of sweet potato tuber (He et al., 2020; Suematsu et al., 2020; Bennett et al., 2021). However, little is known about the effect of LEDs on the physicochemical quality of sweet potato leaves and their metabolic and transcriptome changes. We performed metabolome and transcriptome profiling on sweet potato leaves grown under monochromatic red or blue lights to identify differentially expressed genes and biosynthetic pathways affecting metabolite biosynthesis and their differential accumulation.

## 2 Materials and methods

### 2.1 Growth environment and plant materials

**Figure 1:**
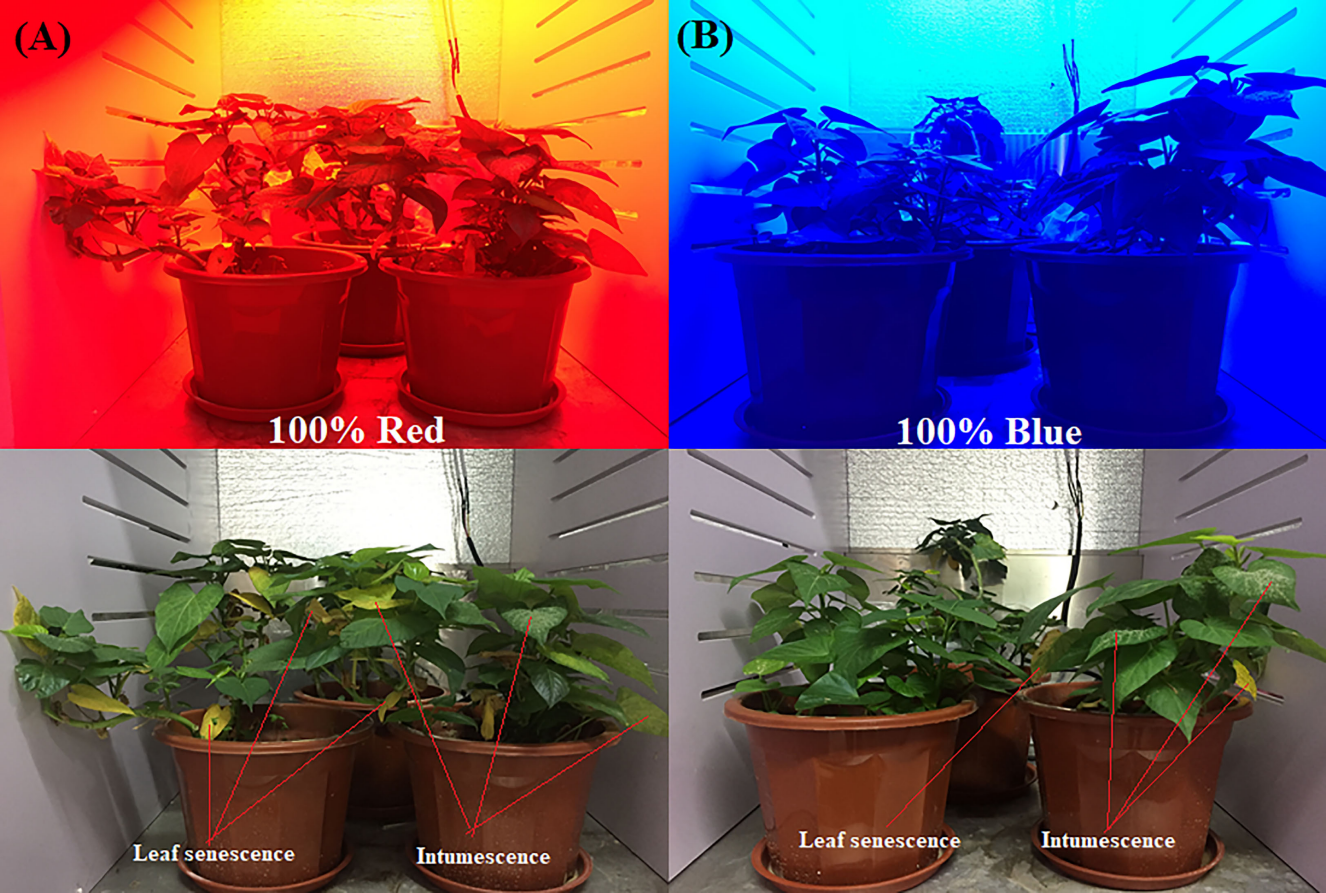
Sweet potato (*I. batatas* (L.) Lam) vines growing under 100% red **(A)** and 100% blue **(B)** LEDs at three weeks after exposure, indicating the degree of leaf senescence and intumescence in both conditions.

The experiment was conducted in a controlled chamber (Talos Technology, Fujian, China) illuminated with monochromatic red [100%, 632 (nm)] or blue [100% 462 (nm)] LEDs (**Figure 1**). Low intensity LEDs with photosynthetic photon flux density [PPFD (50 μmol m^-2^ s^-1^)] were used to induce sweet potato leaf development. The photoperiod (16/8 hrs), temperature (25 ± 2°C), and relative humidity (70 ± 5%) were kept constant. The sweet potato (‘Fushu-18’) vines were harvested from the Key Laboratory of Crop Biotechnology, Fujian Agriculture and Forestry University, China. The lower leaves were removed with disinfected scissors, leaving the fully opened leaves (18/22) to achieve uniformity before transplanting to pots (16 cm width x 14 cm height) filled with a mixture of 750 ± 10 g of organic soil (70% organic matter content, 40% humic acid, 3% NPK, and a pH of 6.5) and clean river sand (1:2). Three pots planted with three vines were assigned to each treatment and replicated three times. The vines were grown for two weeks under ambient conditions (open field with full sunlight exposure) before being transferred to the growth chamber and exposed to red or blue LEDs for three weeks. A nutrient solution (Sara Hyponica, Japan) containing (A%) nitrogen (1.0), phosphoric acid (3.80), alkali (5.50), magnesium oxide (1.0), manganese (0.03), boron (0.05), and nitric acid (3.0) was added to the irrigation water every three days.

### 2.2 Measurement of growth indices

The morphological and growth data were collected from three leaf samples in each replication in the third week after the vines were exposed to blue or red light regimes. The membrane stability index (MSI) was measured using a conductivity meter (Pancellent Inc., China) according to the method described by Dwivedi et al. (2018). The leaf water content (LWC) was estimated following the procedure reported by He and Qin (2020). The leaf area (LA) was estimated with the method earlier reported by Yeshitila and Taye (2016). The length and width of the sixth leaves were marked and measured before and after transfer to the growth chamber. The number of abscised leaves (NAL) and the percentage of leaves with intumescence were calculated using the following equations:


NAL=TNAL/TNL*100

Intumescense(%)=NAL/TNL*100

NAL, TNL, and TNAL are the number of affected leaves, number of whole leaves and total number of abscised leaves, respectively.

### 2.3 Determination of chlorophyll pigments, total soluble sugar, protein, and vitamin C

Chlorophyll pigments were measured spectrophotometrically (T6 series, Persee Analytics, Inc. Auburn, CA 95603) according to Lichtenthaler and Babani (2022). The biochemical contents were measured at three weeks after exposure to light regimes. Three middle leaves from each replication were removed, ground, and frozen in liquid nitrogen until use. The total soluble sugar, protein, and vitamin C were measured from the frozen leaf samples (0.2 and 1.0 g). The anthrone colorimetric method was employed to determine soluble sugar (Ahsen et al., 2019). Coomassie blue G250 was used to estimate the total soluble protein content (Ahsen et al., 2019). The titration method was used to estimate the vitamin C content (Kui et al., 2020). The absorbance of the samples (sucrose 620 nm and protein 595 nm) was measured with a UV−VIS spectrometer (T6 series., Persee Analytics, Inc. Auburn, CA 95603).

### 2.4 Total flavonoids, phenolics, and antioxidant scavenging activity

The total flavonoid content (TFC) was determined according to a method by Wei et al. (2019). Three middle leaves were collected from each replication and rutin standard solution was used for the calibration curve. The total phenolic content (TPC) was estimated using the Folin-Ciocalteu method of Alara et al. (2020), and garlic acid (0.1 mg ml^-1^) was used in the standard curve. The total antioxidant activity was estimated using the 2,2-diphenyl-1-picrylhydrazyl (DPPH) assay (Guo et al., 2017).

### 2.5 Metabolome analyses of sweet potato leaves

Freshly opened leaves (fully opened) were harvested and used for metabolome profiling. A 0.5 g sample from each replicate was weighed, frozen in liquid nitrogen and subsequently stored in a -80°C refrigerator. The frozen samples were crushed with a mixer mill (MM 400, Retsch) and zirconia beads for 1.5 minutes at 30 Hz. The lyophilized powder (100 mg) was dissolved in 1.2 mL of methanol (70%) solution, vortexed six times for 30 secs every 30 mins, and placed in a 4°C refrigerator overnight. The samples were then centrifuged for ten minutes at 12,000 rpm, and the extracts were filtered (SCAA-104, 0.22 μm pore size; ANPEL, Shanghai, China; http://anpel.com.cn/) before ultra-performance liquid chromatography-Mass spectroscopy (UPLC−MS/MS) analysis. All reagents used in the metabolic analyses were sourced from Merck (Merck, China), except the standard (BioBio/Sigma−Aldrich, China).

Metabolite extraction, detection, and quantification were performed by the Wuhan MetWare Biotechnology Co., Ltd., China (https://metware.cn) according to the method described by Fraga et al. (2010). The sample extracts were analyzed using a UPLC−ESI−MS/MS system (UPLC, SHIMADZU Nexera X2, https://shimadzu.com.cn/; MS, Applied Biosystems 6500 Q TRAP). The analytical conditions were as follows: UPLC column, Agilent SB-C18 (1.8 µm, 100 mm * 2.1 mm). The mobile phase consisted of distilled water with 0.1% formic acid, solvents A and B, and 0.1% formic acid with acetonitrile. The sample analyses were performed with a gradient program that used 95% A and 5% B as starting conditions. Within the first nine minutes, a linear gradient of 5% A and 95% B was programmed, and a composition of 5% A and 95% B was maintained for 1 minute. Then, a composition of 95% A and 5.0% B was adjusted for 1.1 minutes and held for 2.9 minutes. The flow velocity was kept at 0.35 mL per minute, and the column temperature was maintained at 40°C. An injection volume of 2 μL was used, and the effluent was connected to the ESI-triple quadrupole-linear ion trap (QTRAP)-MS. Three biological replicates (young fresh leaves) were collected and analyzed for each sample, and the sample extract mixture was used for quality checks.

#### 2.5.1 Differentially accumulated metabolites and KEGG annotation/enrichment analyses

The metabolites detected were screened with stringent threshold: absolute log_2_FC (fold change) ≥ 1 and variable importance in projection (VIP) ≥ 1 to identify differentially accumulated metabolites (DAMs). The VIP values were obtained from the Orthogonal Projections to Latent Structures-Discriminant Analysis (OPLS-DA) result with score and permutation plots. The data were generated using the R package *MetaboAnalyst*, log2-transformed (log_2_), and mean-centered before OPLS-DA. To avoid overfitting, a permutation test (200 permutations) was performed on the data (Hua et al., 2021; Xu et al., 2022). For the Kyoto Encyclopedia of Genes and Genomes (KEGG) annotation, the detected metabolites were annotated using the KEGG compound database (http://kegg.jp/kegg/compound/) and subsequently mapped to the KEGG pathway database (http://kegg.jp/kegg/pathway.html). The pathways with significantly regulated metabolites mapped were then subjected to metabolite set enrichment analysis (MSEA), and their significance was evaluated by the hypergeometric test using p-values (*p< 0.05*).

### 2.6 Transcriptome (RNA-Seq) analysis

Freshly opened leaves (0.5 g) were harvested from each replicate, immediately stored at -80°C and sent to the Biomarker Technologies Laboratory (http://en.biomarker.com.cn/) for analysis. The ribonucleic acid (RNA) concentration and purity were measured using a NanoDrop 2000 (Thermo Fisher Scientific, Wilmington, DE). The RNA integrity was assessed using the RNA Nano 6000 Assay Kit of the Agilent Bioanalyzer 2100 system (Agilent Technologies, CA, USA). A total of 1 μg of RNA per sample was used as input material for the RNA sample preparations. According to the manufacturer’s recommendations, sequencing libraries were generated using the NEBNext UltraTM RNA Library Prep Kit for Illumina (NEB, USA). Then, PCR was performed with Phusion High-Fidelity DNA polymerase, Universal PCR primers, and Index (X) Primer. Finally, the PCR products were purified (AMPure XP system), and library quality was assessed on the Agilent Bioanalyzer 2100 system. The clustering of the index-coded samples was performed on a cBot Cluster Generation System using TruSeq PE Cluster Kit v4-cBot-HS (Illumina). Raw FASTQ format data were first processed through in-house Perl scripts to obtain clean data by removing reads containing adapter, poly-N, and low-quality reads. All downstream analyses were based on clean, high-quality data. After data processing, raw sequences were transformed into clean reads and mapped to the reference genome. Only reads with a perfect match or one mismatch were further analyzed and annotated based on the reference genome using HISAT2 software.

#### 2.6.1 Gene functional annotation, quantification, and enrichment

The gene functions were annotated based on the National Center for Bioinformatic Information (NCBI) nonredundant protein sequences (Nr), NCBI nonredundant nucleotide sequences (Nt), protein family (Pfam), clusters of orthologous groups of proteins (KOG/COG), Swiss-Prot; KEGG ortholog database (KO), and gene ontology (GO) databases. Differential expression analysis of two conditions/groups (blue vs red lights) was performed using *DESeq2*. The resulting P values were adjusted using Benjamini and Hochberg’s approach (Benjamini and Hochberg, 1995). False discovery rate (FDR)< 0.01 and fold change ≥ 2 were set as the thresholds for significant differential expression. Gene Ontology (GO) enrichment analysis of the differentially expressed genes (DEGs) was performed by the *GOseq* R package based on Wallenius noncentral hypergeometric distribution (Young et al., 2010). We used the KEGG (Kanehisa et al., 2008) database to understand the functions and utilities of the biological and molecular datasets generated by genome sequencing (http://www.genome.jp/kegg/). The KOBAS software (Mao et al., 2005) was used to generate the statistical enrichment of DEGs in the KEGG pathways. The sequences of the DEGs were blasted (blastx) to the genome of related species (the protein−protein interaction (PPI) in the STRING database: http://string-db.org/) to obtain the predicted PPI of these DEGs. Then, the PPIs of these DEGs were visualized in Cytoscape (Shannon et al., 2003).

To validate the reliability of the transcriptome data, quantitative real-time polymerase chain reaction (qRT−PCR) was performed with nine randomly selected genes. The qRT−PCR gene-specific primers (**Table S1**) were designed using Primer Express software (v3.0, Applied Biosystems). The *ACTIN* gene (EU250003.1) was used as the internal reference gene to normalize the gene expression values. The 2^-ΔCT^ method (Livak and Schmittgen, 2001) was used to calculate the relative gene expression levels.

### 2.7 Statistical analyses

The physiological and biochemical data obtained were analyzed in Microsoft Excel for the means and standard errors. Student’s *t* test was used to evaluate the effect of red and blue lights on sweet potato leaves. Unsupervised principal component analysis (PCA) was performed using the statistical function *prcomp* in R (www.r-project.org). The data were unit variance scaled before the unsupervised PCA. Hierarchical cluster analysis (HCA) was performed by *pheatmap* (Kolde, 2012), and Pearson correlation coefficients (r) between the samples were calculated by the *corrplot* package in R (Wei et al., 2017).

## 3 Results

### 3.1 Effect of red and blue LEDs on physiological attributes and bioactive compounds in sweet potato leaves grown in a controlled environment

This study evaluated the effects of light on the physicochemical and growth parameters (**Table 1**) of sweet potato leaves grown under low intensity monochromatic red or blue LEDs. The Light quality significantly affected the quality of the sweet potato leaves (**Figure 1**) after three weeks of exposure to the red and blue LEDs. Growing sweet potato under blue light resulted in higher physiological indices (increased membrane stability (93 ± 0.33% vs 90 ± 1.06%), fresh leaf (0.50 ± 0.04g vs 0.32 ± 0.01g), dry weights (0.05 ± 0.01g vs 0.02 ± 0.00g), leaf area (165.1 cm^2^ ± 1.84 vs 162.4 ± 0.10 cm^2^), number of abscised leaves (25 ± 2.4% vs 47 ± 2.7%) and intumescence (13 ± 1.6% vs 25 ± 1.8%), in the leaves of the sweet potatoes, except for the leaf water content (89 ± 0.53% vs 95 ± 1.06%) relative to the red LEDs (**Table 1**; **Figure S1**). The vines grown under red LEDs showed an increase in biochemical activity; thus, they produced more chlorophyll *a*, *b*, and total carotenoid contents than those grown under blue LEDs. Conversely, the vines grown under blue LEDs recorded higher soluble protein contents, total phenols, flavonoids, and 2,2-diphenyl-1-picrylhydrazyl antioxidant activity (**Table 1**).

**Table 1:** Influence of red and blue LEDs on physiological attributes and bioactive compounds of sweet potato (*I. batatas* (L.) Lam) leaves.

Parameter	Treatments		
	Blue light (100%)	Red light (100%)	*P-value*
Physiological attributes			
Membrane stability index (%)	93 ± 0.33	90 ± 1.06	*
Leaf water content (%)	89 ± 0.53	95 ± 1.06	*
Leaf fresh weight (g)	0.50 ± 0.04	0.32 ± 0.01	*
Leaf dry weight (g)	0.05 ± 0.01	0.02 ± 0.00	**
Leaf area (cm^2^)	165.1 ± 1.84	162.4 ± 0.10	NS
Number of abscise leaves (%)	25 ± 2.4	47 ± 2.7	**
Intumescence (%)	13 ± 1.6	25 ± 1.8	**
Bioactive compounds			
Chlorophyll (mg g^-1^)	16.47 ± 0.60	18.41 ± 0.66	NS
Chlorophyll (mg g^-1^)	5.33 ± 0.29	7.26 ± 0.37	**
Total Chlorophyl (mg g^-1^)	21.80 ± 0.89	25.67 ± 1.03	**
Carotenoids (mg g^-1^)	3.46 ± 0.12	4.07 ± 0.15	**
Total soluble sugar (%)	0.5 ± 0.02	0.7 ± 0.02	**
Total soluble protein (%)	1.3 ± 0.09	0.7 ± 0.01	**
Vitamin C (mg g^-1^)	10.4 ± 0.38	19.83 ± 0.77	***
Total Phenolics (mg g^-1^)	259.3 ± 8.61	199.8 ± 1.61	**
Total flavonoids (mg g^-1^)	54.7 ± 0.95	40.1 ± 0.64	***
Antioxidant scavenging activity	94.9 ± 0.14	91.1 ± 0.58	**

Means were separated by student t-test. The mean ± standard error with *, **, and *** indicates significance at P<0.05, P<0.01 and P<0.001, respectively. NS indicates no significant difference.

### 3.2 Metabolome profiles and analysis of differentially accumulated metabolites in sweet potato leaves grown under red and blue LEDs

**Figure 2:**
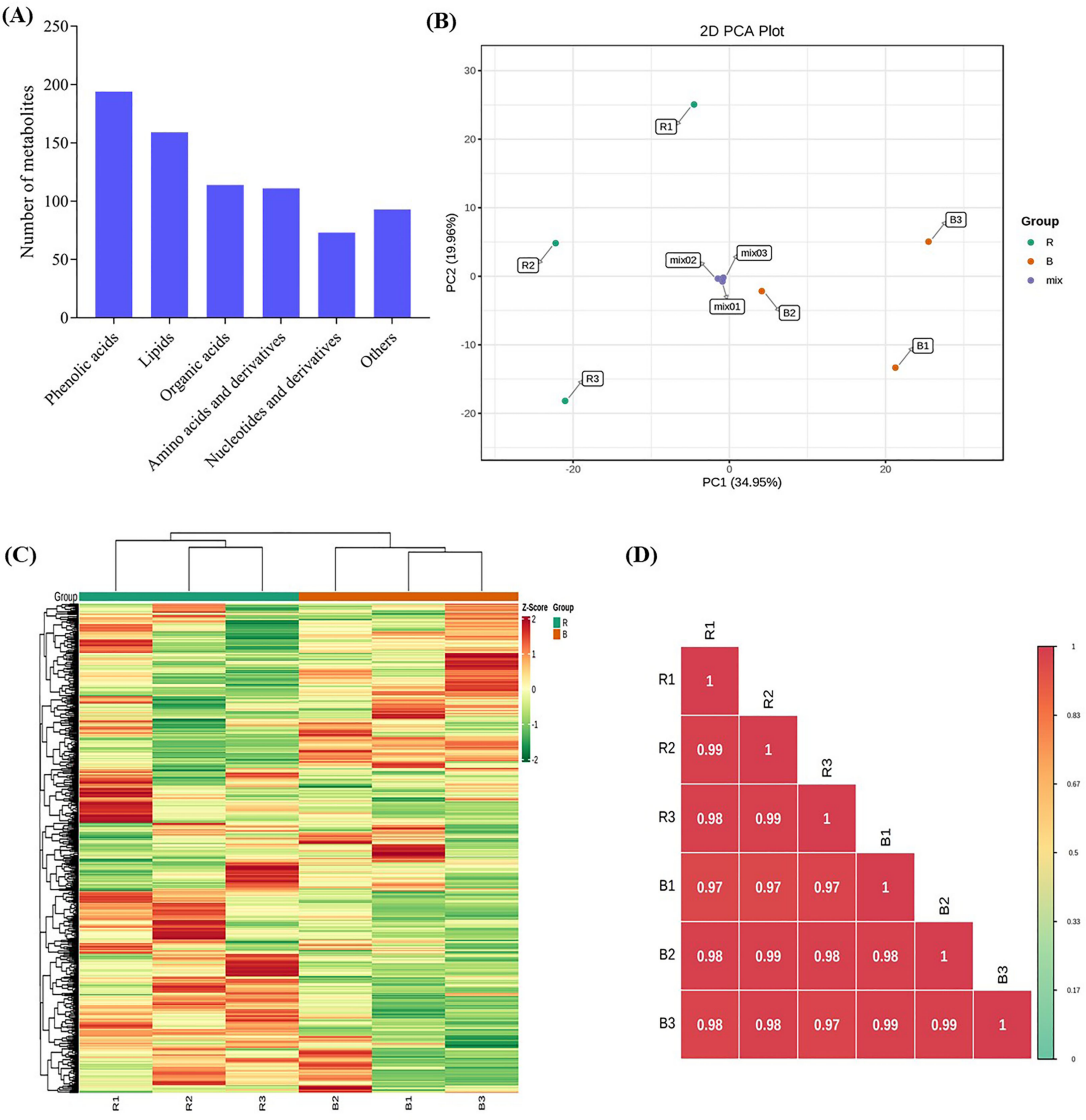
Metabolites detected in the sweet potato (*I. batatas* (L.) Lam) leaves growing under red (R) and blue **(B)** LEDs. L1-100% is blue (100%), and L2-100% is red (100%) LEDs. All analyses were done in triplicates, thus R (R1-R3) and B (B1-B3). **(A)** Classes of compounds detected. **(B)** Principal component analysis plot based on two principal components axes. **(C)** Hierarchical heatmap clustering. **(D)** Pearson correlation coefficients plot of differentially accumulated metabolites.

The sweet potato vines were produced from a controlled growth chamber to investigate the effect of blue and red LEDs on the sweet potato leaves. Representative photographs of vine growth conditions and appearance after three weeks of light treatments are presented in **Figure 1**; **S1**. UPLC−ESI−MS/MS was used to analyze the key metabolites in sweet potato leaves produced under red or blue LEDs. In all, 744 compounds were detected, comprising 194 phenolic acids, 159 lipids (glycerol ester, free fatty acids, PC, sphingolipids, LPC and LPE), 114 organic acids, 111 amino acids and derivatives, 73 nucleotides and derivatives, and 93 other compounds (saccharides, alcohols, and vitamins) (**Table S2**; **Figure 2A**). The data similarity and dissimilarity were evaluated using principal component analysis (PCA), hierarchical clustering (HCA) and correlation analyses using the ion intensities of the measured metabolites. The PCA revealed that the first two PCA axes could explain 54.91% of the variability between samples from the red and blue LED treatments. The clustering showed relatively distinct variation between the treatments alone and mixtures (**Figure 2B**). The PCA, together with HCA and high correlation coefficients (**Figures 2B–D**) suggest a high reproducibility and repeatability of the samples from the same light treatment in this study, lending credence to our dataset’s reliability for downstream analyses.

**Figure 3:**
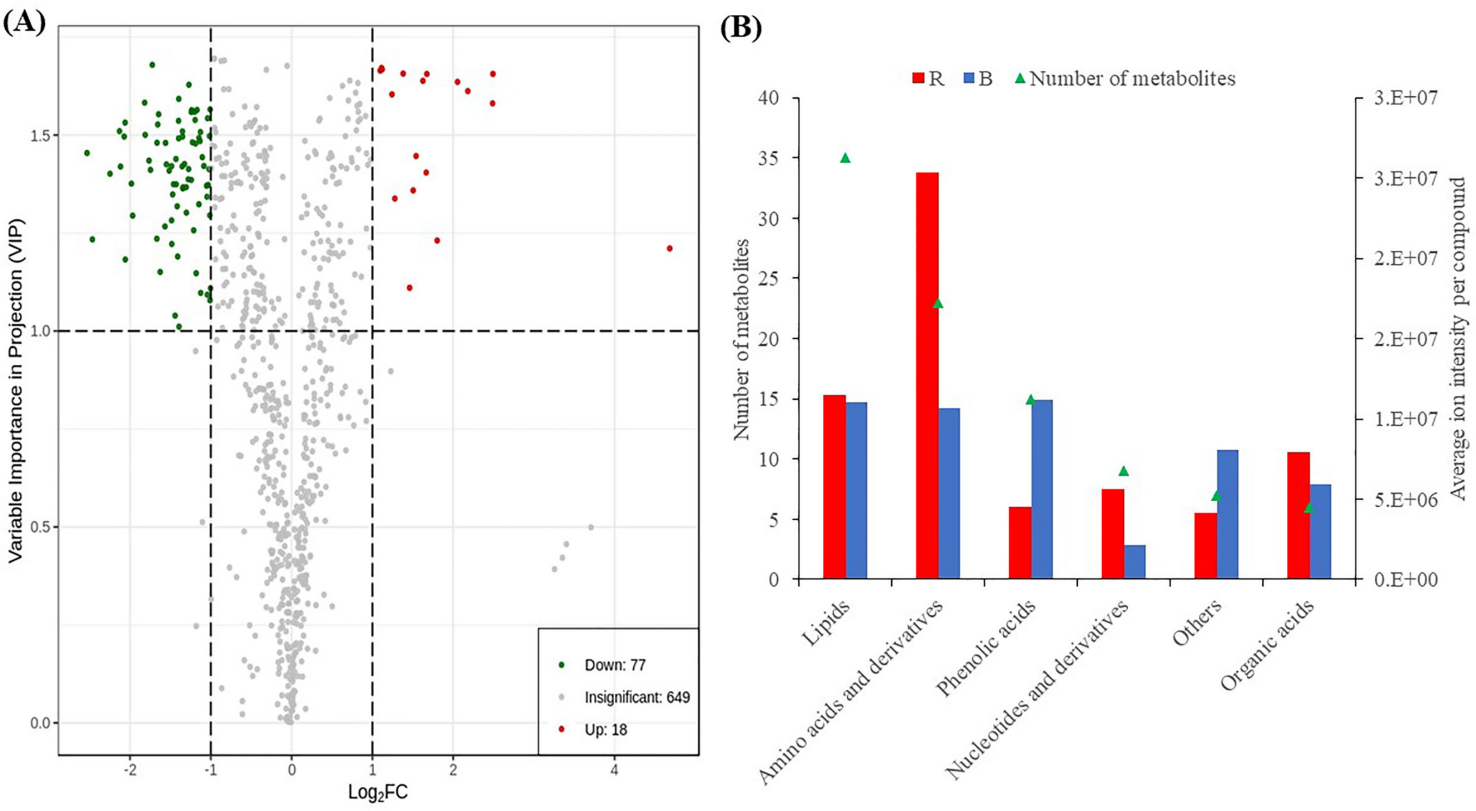
Differentially accumulated metabolites detected between sweet potato leaves under red (R) and blue **(B)** LEDs. **(A)** Volcano plot of metabolites. The red and blue points indicate the up-regulated and down-regulated metabolites, respectively, and the gray area represents metabolites detected but not significant. **(B)** Classes of compounds detected as DAMs. The primary Y-axis represents the number of metabolites, while secondary Y-axis represents the classes of compounds.

**Figure 4:**
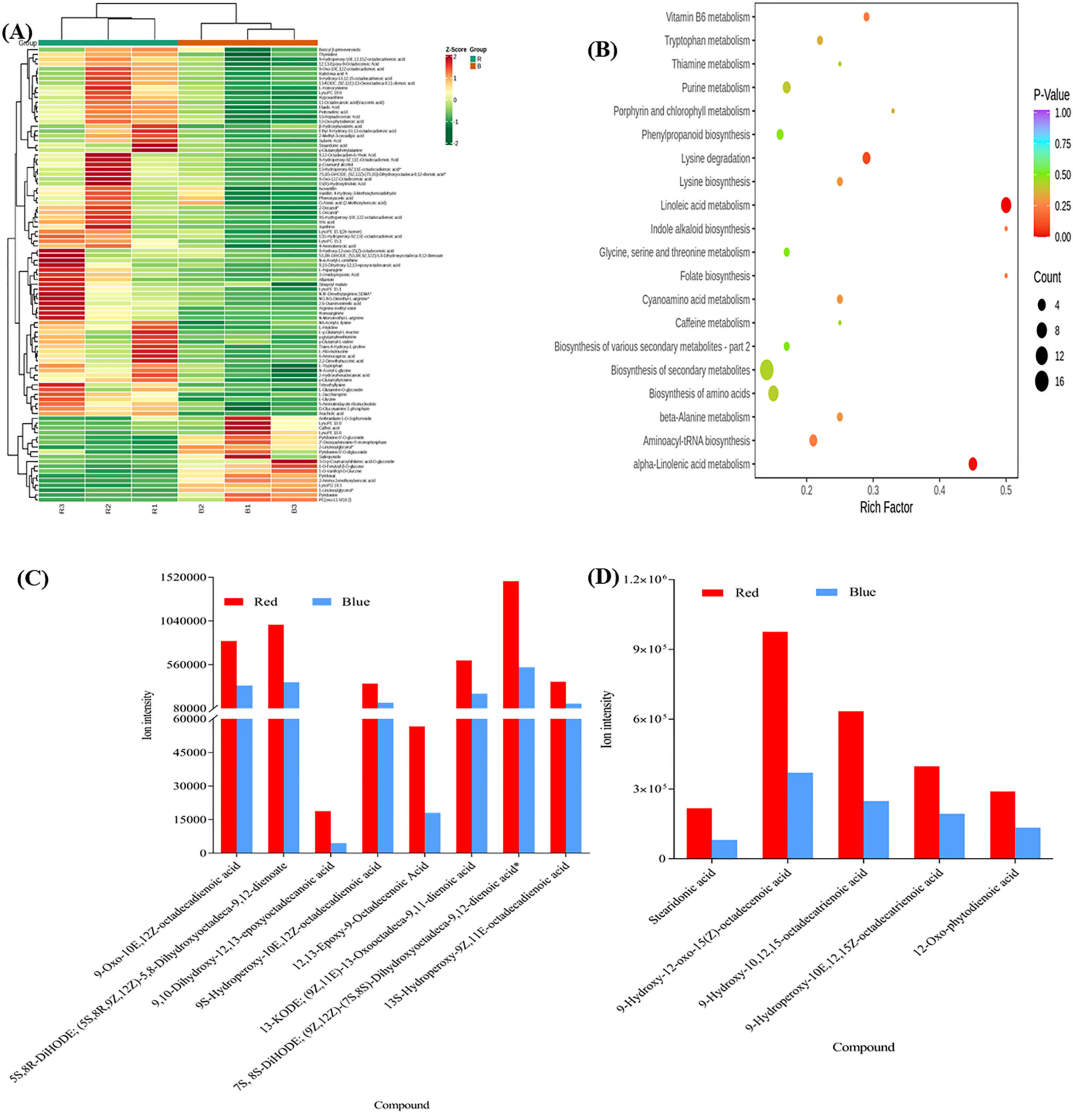
**(A)** The Differential metabolites clustering heatmap (R vs B) of sweet potato (*I. batatas* (L.) Lam) leaves grown under red (R) and blue **(B)** LEDs. The bar above the heatmap corresponds to the sample clustering (group). The dendrogram on the left side of the heatmap represents the differential metabolite clustering. The annotation bar on the left side of the heat map corresponds to the first-level classification (Class) of the compounds, and different colors represent different compound categories. **(B)** The Kyoto encyclopedia of genes and genomes (KEGG) pathway enrichment analysis bubble plot of differentially accumulated metabolites. Each row represents a KEGG pathway. The color of the dots represents the p-value, and the size of the bubbles represents the number of differential metabolites annotated in that pathway. Those underlined had p-values< *0.05*, and the KEGG pathway significantly (p< 0.05) enriched with differentially accumulated metabolites in the sweet potato leaves. **(C)** Linoleic acid metabolism. **(D)** Alpha-Linolenic acid metabolism.

To identify differentially accumulated metabolites, we applied stringent screening criteria (VIP ≥ 1 and absolute log_2_FC ≥ 1) were applied and detected 95 DAMs, comprising 77 and 18 down- and up-regulated metabolites, respectively (**Figure 3A**; **Table S3**), implying that red LED treatment induced increased accumulation of metabolites compared with blue LEDs. The DAMs consisted of 35 lipids, 23 amino acids and derivatives, 15 phenolic acids, 9 nucleotides and derivatives, 6 organic acids and 7 other compounds (**Figure 3B**). Only phenolic acids and other compounds were accumulated higher under blue light than red light conditions. All six classes of compounds showed contrasting accumulation levels between sweet potato vines evaluated under red and blue LEDs (**Figure 4A**).

#### 3.2.1 Comparative analysis of changes in metabolites in the sweet potato leaves under red and blue LED treatment

**Figure 5:**
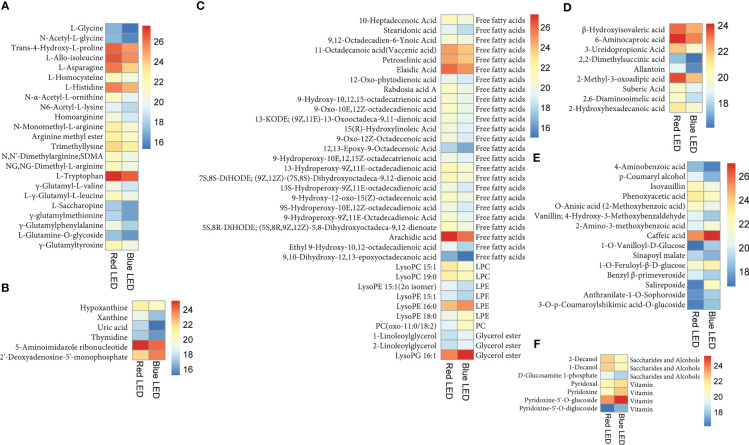
Heatmap of accumulation of different classes of compounds among the 95 differentially accumulated metabolites (DAMs) based on log2 transformed ion intensity of each compound between red (R) and blue **(B)** LEDs. **(A)** Amino acids and derivatives. **(B)** Nucleotides and derivatives. **(C)** Lipids. **(D)** Organic acids. **(E)** Phenolic acids. **(F)** Other classes. Each row represents one metabolite on the left-hand side of each heatmap. While in the case of lipids **(C)** and others **(F)** have sub-classes of compounds on the right-hand side of the heatmaps. The color gradients are shown on the right-hand side of each heatmap.

The light quality caused significant variations in the DAMs (**Figure 5**). Red light significantly increased the accumulation of 23 amino acids and derivatives including eight essential amino acids (**Figure 5A**). The essential amino acids consist of L-allo-isoleucine, L-histidine, N6-acetyl-L-lysine, L-tryptophan, γ-glutamyl-L-valine, L-γ-glutamyl-L-leucine, γ-glutamylmethionine and γ-glutamylphenylalanine. The remaining 15 compounds comprise non-essential amino acids. In addition to amino acids and derivatives, six nucleotides and derivatives accumulated differentially in potato leaves treated with red and blue LEDs. Of these, only 2’-deoxyadenosine-5’-monophosphate accumulated higher in blue light than in red light, while the remaining five all accumulated highly under red LEDs (**Figure 5B**).

Light quality significantly altered lipid composition and abundance in the sweet potato leaves (**Figure 5C**). Red light sharply increased the abundance of 25 free fatty acids including stearidonic acid (SDA) and arachidic acid as well as two lysophosphatidylcholine (lysoPC) (lysoPC 15:1 and lysoPC 19:0) and two lysophosphatidylethanolamine (lysoPE) (lysoPE 15:1(2n isomer) and lysoPE 15:1) relative to blue light. Interestingly, three other lipids (lysoPE 16:0, lysoPE 18:0 and PC(oxo-11:0/18:2)) and three glycerol ester (1-linoleoylglycerol, 2-linoleoylglycerol and lysoPG 16:1) accumulated higher under blue LEDs than in red LEDs (**Figure 5C**). Red light increased the abundance of nine organic acids, including suberic acid, 2-hydroxyhexadecanoic acid and 6-aminocaproic acid compared to sweet potato leaves cultivated under blue LEDs (**Figure 5D**). In addition, phenolic acid composition in the sweet potato leaves varied significantly under the two light treatments. Among the phenolic acid compounds, eight compounds (such as 4-aminobenzoic acid, p-coumaryl alcohol and isovanillin) increased highly under red LEDs, while seven others including caffeic acid increased highly under blue light (**Figure 5E**).

Also, the light quality altered saccharide and alcohol, and vitamin composition in the sweet potato leaves (**Figure 5F**). Three saccharide and alcohol; 2-decanol, 1-decanol and D-glucosamine 1-phosphate accumulated higher under red light than blue light (**Figure 5F**). While four vitamins mainly vitamin B6 (pyridoxal, pyridoxine, pyridoxine-5’-O-glucoside and pyridoxine-5’-O-diglucoside) accumulated highly under blue LEDs (**Figure 5F**). Altogether, the above results indicate that the light quality altered the composition of metabolites in the sweet potato leaves including essential amino acids and other compounds of nutritional importance to humans.

#### 3.2.2 KEGG pathway enrichment analyses among the DAMs

The DAMs detected were subjected to KEGG pathway enrichment analyses (*P* value< *0.05*). We observed that alpha-linoleic acid metabolism (ko00592) and linolenic acid metabolism (ko00591) were the two most significantly enriched pathways, with 9 and 5 DAMs, respectively (**Table S4**; **Figure 4B**), indicating that the light quality caused differential accumulation of metabolites in these two metabolic pathways. The metabolites involved in linolenic acid metabolism (ko00591) increased by 59.45% for 9S-hydroperoxy-10E,12Z-octadecadienoic acid and 76% for 9,10-dihydroxy-12,13-epoxyoctadecanoic acid in sweet potato leaves grown under red LEDs compared to blue LEDs (**Figure 4C**). Similarly, red light treatment increased stearidonic acid, 9-hydroxy-12-oxo-15(Z)-octadecenoic acid, 9-hydroxy-10,12,15-octadecatrienoic acid, 9-hydroperoxy-10E,12,15Z-octadecatrienoic acid and 12-oxo-phytodienoic acid by 62.54, 61.94, 60.89, 53.53 and 51.36%, respectively, compared with blue LED treatment (**Figure 4D**). These results suggest that red light treatment could be exploited to increase the metabolome profile in sweet potato leaves for food and nutritional purposes.

### 3.3 Transcriptome profiling of sweet potato leaves

The transcriptome analysis generated 40.08 Gb of clean data **Table S5**). At least 5.97 Gb clean data were generated for each sample, with a minimum of 94.27% clean data based on the Q30 quality score **Table S5**). Clean reads of each sample were mapped to the sweet potato reference genome database (http://public-genomes-ngs.molgen.mpg.de/sweetpotato/). The mapping ratio ranged from 75.49% to 76.50% (**Table S5**). Then, all expressed transcripts were analyzed using pairwise comparisons of the two samples. However, prediction of alternative splicing, gene structure optimization, and novel gene discovery were made from the mapping results. A total of 17,995 genes were discovered, and 14,039 novel genes were annotated with putative functions.

#### 3.3.1 Analysis of differentially expressed genes and functional annotation

We further subjected the expressed genes to differential analysis with a stringent threshold of FDR< 0.01 and FC ≥ 2. From this analysis, a total of 615 DEGs were detected, with 510 down-regulated (higher expression in blue LED-treated sweet potato than red LED) and 105 up-regulated (higher expression in red LED-treated sweet potato than blue LED) DEGs (**Figure S2**). These results indicate that LED treatment of sweet potato leaves causes transcriptional alterations.

**Figure 6:**
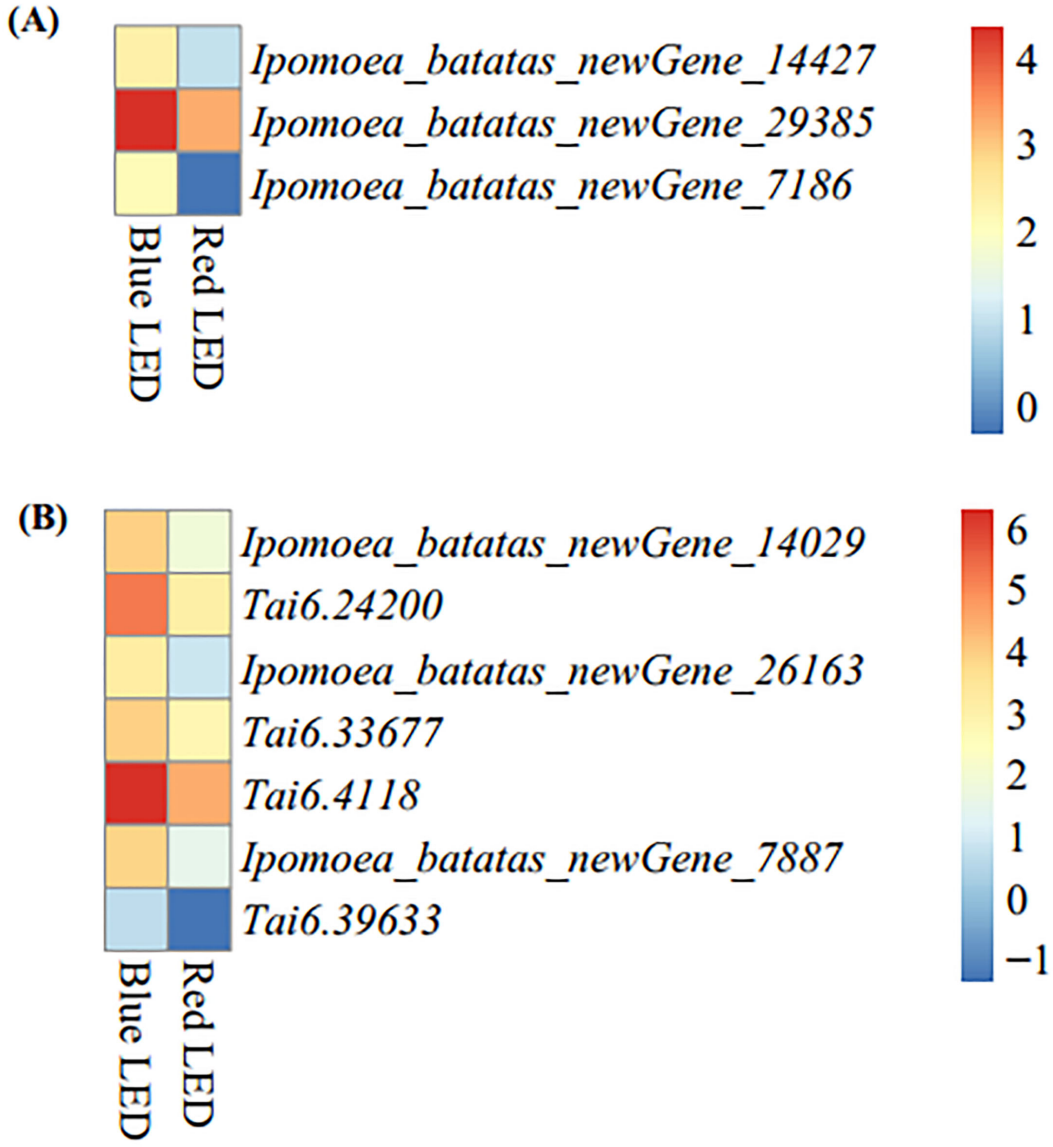
Heatmap of differentially expressed genes enriched in nutritionally-related KEGG pathways based log2 transformation of fragments kilobase of exon per million fragments mapped (FPKM) of sweet potato (*Ipomoea batatas* (L.) Lam) leaves grown with red and blue LEDs. **(A)** Anthocyanin biosynthesis with three structural genes (*Ipomoea_batatas_newGene_14427*, *Ipomoea_batatas_newGene_29385* and *Ipomoea_batatas_newGene_7186*) encode for anthocyanidin 3-O-glucosyltransferase [EC:24.1.115], **Figure 5**. **(B)** Carotenoid biosynthesis with seven structural genes, including two genes (*Ipomoea_batatas_newGene_14029* and *Tai6.24200*) encode carotenoid isomerase (crtH,crtISO) [EC:5.2.1.13], one gene (*Ipomoea_batatas_newGene_26163*) encode beta-carotene 9-cis-all-trans isomerase [EC:5.2.1.14], one gene (*Tai6.33677*) encode for zeaxanthin [EC:1.14.1521], one gene (*Tai6.4118*) encode for antheraxanthin [EC:1.23.5.1] and two genes (*Ipomoea_batatas_newGene_7887* and *Tai6.39633*) encode for abscisate [EC:1.14.14137] on **Supplementary Figure B**.

With the nutritional importance of sweet potato leaves to human nutrition, we mined DEGs enriched in the nutritionally-rich anthocyanin and carotenoid biosynthetic pathways from the KEGG analyses (**Figure S3**). The blue light treatment resulted in higher expression of anthocyanin biosynthetic (ko00942) genes (*Ipomoea_batatas_newGene_14427*, *Ipomoea_batatas_newGene_29385* and *Ipomoea_batatas_newGene_7186*) than the red light treatment (**Figure 6A**). These three genes encode anthocyanidin 3-O-glucosyltransferase [EC:2.4.1.115] (**Figure S4**). These genes could be targeted for functional validation and verification to unravel their regulatory role in improving the nutritional composition of sweet potato leaves.

Additionally, seven carotenoid biosynthetic (ko00906) genes (*Ipomoea_batatas_newGene_14029*, *Tai6.24200, Ipomoea_batatas_newGene_26163*, *Tai6.33677, Tai6.4118, Ipomoea_batatas_newGene_7887* and *Tai6.39633*) were expressed at higher levels in blue LED-treated leaves than in red LED-treated leaves (**Figure 6B**). These genes encode different enzymes (carotenoid isomerase (crtH, crtISO) [EC:5.2.1.13], beta-carotene 9-cis-all-trans isomerase [EC:5.2.1.14], zeaxanthin [EC:1.14.1521], antheraxanthin [EC:1.23.5.1] and abscisate [EC:1.14.14137]) on the carotenoid biosynthetic pathways (**Figure S5**). These results suggest that blue LED treatment could be used to improve the nutritional value of sweet potato leaves for human and animal consumption.

**Figure 7:**
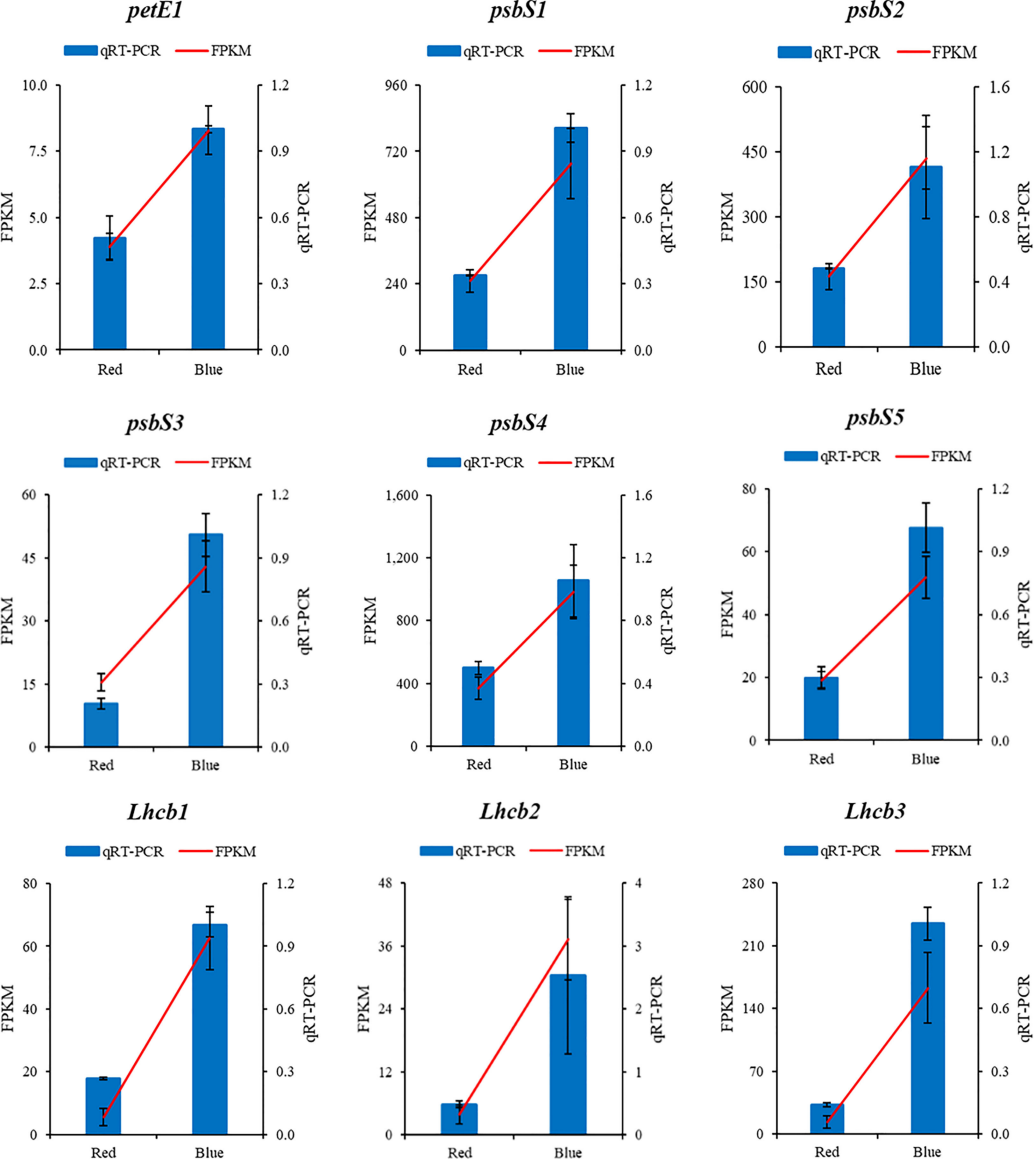
The qPCR validation of the RNA sequencing results of photosynthesis and photosynthesis-antenna proteins genes in sweet potato (I. batatas (L.) Lam) leaves grown under red and blue LEDs.

To validate the transcriptome data, total RNA from the same two samples used for RNA sequencing was used as a template for qPCR. The qRT−PCR validation results revealed high similarity to the RNA sequences identified with the fragments per kilobase of exon per million mapped fragments (FPKM) values from the sequencing results under the red and blue light treatments (**Figure 7**).

## 4 Discussion

Light conditions substantially impact sweet potato leaves, which differ in their shape and metabolic profiles (Lv et al., 2021). Commercial horticultural producers have been experimenting with red and blue LEDs in their production systems, as these lights are utilized to produce high-quality horticultural products (Bantis et al., 2018). Leaves are the principal light collectors during photosynthesis, which impacts their quality and metabolite accumulation (Yue et al., 2021). There was less abscission and intumescence in the sweet potato leaves grown under blue LEDs than in those produced under red LEDs. Ornamental sweet potatoes and tomatoes have been found to have a lower frequency of anatomical disorders when exposed to blue light (Craver et al., 2014; Eguchi et al., 2016). Intumescence formation is genetically controlled in various plant species and significantly affects photosynthesis. It also negatively impacts plant tissues by inducing chlorosis, senescence, abscission and downward curling of leaves (Williams et al., 2016). However, red light has long been thought to delay leaf senescence (Sakuraba, 2021). Senescence in grapes has also been shown to be delayed under red light exposure (Wang et al., 2016b). In this study, harvested leaves were more robust under blue light and had higher fresh and dry weights. Zheng and Van Labeke (2017) reported that light intensity and quality affect biomass accumulation in ornamental plants. In sweet potato leaves grown under red LEDs, we found significant concentrations of chlorophyll *a* and *b*, total chlorophyll, and carotenoid pigments (**Table 1**). In contrast, Yousef et al. (2021) found a lower pigment concentration in two tomato seedlings grown under monochromatic red lights, indicating that PSII had been altered and may be compromised in functionality. However, earlier studies showed that combining blue and red light was more successful in fostering plant growth than monochromatic lighting (Liang et al., 2021). In addition, the photosynthetic rate is increased by red light, which affects the transport of carbohydrates from leaves to roots (Zhong et al., 2021).

LEDs have been used as substitutes for conventional light sources in controlled agricultural environments (Yousef et al., 2021). They emit specific wavelengths of light suitable for photosynthesis and photomorphogenesis (Bantis et al., 2018). In this study, leaves grown under blue LEDs showed enhanced accumulation of total phenols and flavonoids and increased antioxidant scavenging activity (**Table 1**). Tuladhar et al. (2021) reported that stressed plants showed increased biosynthesis of several compounds. In addition, the effects of red, blue, and far-red LEDs on phenol and flavonoid accumulation in diverse plant species have been well documented (Amoozgar et al., 2017; Nam et al., 2018). The roles of monochromatic red and blue LEDs depend on certain factors that need to be optimized. Studies have shown that blue and red light stimulate the activity of enzymes in the phenylpropanoid and shikimate pathways, including chalcone isomerase and synthases, leucoanthocyanidin dioxygenase, flavonol synthase, dihydroflavonol 4-reductase and stilbene (Gam et al., 2020), and phenylalanine ammonia-lyase (Wilawan et al., 2019). Light can affect plant total phenol and flavonoid contents by promoting the expression of genes involved in the biosynthesis of secondary compounds (Gam et al., 2020).

The identification of plant metabolites is an essential step for crop improvement. Therefore, it is vital to study key agronomic traits of crops. The UPLC−MS/MS was used in this study to analyze the metabolites in sweet potato leaves grown under red and blue LEDs. The detected metabolites based on their ion intensities grouped together biological replicates of a sample in the hierarchical heatmap clustering (**Figure 2B**); however, biological replicates of each sample, either red or blue LED clustered together with some discrepancies in PCA (**Figure 2A**). To overcome the discrepancies in the groupings seen in PCA and hierarchical heatmap clustering (**Figures 2B, C**), the DAMs and other downstream analyses were done based on average ion intensities of metabolites of the three biological repeats. The metabolite results showed that red light increased the abundance of metabolites in sweet potato leaves compared to sweet potato leaves grown under blue LEDs (**Figure 4A**). Similarly, red light has been shown to increase the content of amino acids in lettuce grown under red LEDs (Miyagi et al., 2017). In this study, red light significantly increased the accumulation of 23 amino acids and derivatives including eight essential amino acids. In addition, Hashim et al. (2021) reported that LEDs are useful in eliciting essential metabolites in plants for the nutrition and pharmaceutical industries. Besides, red light increased the abundance of 25 free fatty acids relative to blue LEDs. It has been reported that SDA acid is largely supplied to humans from marine sources (Banz et al., 2012), however; our results suggest that red light improve the abundance of SDA. This could serve as sustainable alternative means to curb type II diabetes mellitus (Banz et al., 2012). Red light increased the abundance of nine organic acid compounds in the sweet potato leaves. However, studies have shown that caffeic acid is found in abundance in many plants and foods, such as apples, potato, coffee and red wine. Coffee is the main source of caffeic acid in the diet. It is reported to have antioxidant and anti-inflammatory effects as well have potential to improve the immune system in humans (Zielińska et al., 2021).

Both red and blue lights significantly affected the metabolites detected in the sweet potato leaves (**Table 1**, **Figure 4**). A total of 744 metabolites were identified, with leaves grown under red lights appearing more stressed and accumulating more metabolites than leaves produced under blue LEDs (**Table S2**). However, the total phenols and flavonoids were higher in leaves grown under blue light (**Table 1**). Simultaneously, most of the metabolites were induced and markedly accumulated under red light conditions (**Figure 3A**) as metabolite profiles are affected by differences in plant growth stage (Tamura et al., 2018; Wang et al., 2018). Consistent with our findings, previous studies have shown that red light increases the accumulation of phenolic acids and flavonoids in *M. communis* (Cioć et al., 2018) and *Silybum marianum* L. (Younas et al., 2018), while high levels of phenolics, flavonoids, and antioxidants were reported in tomato seedlings grown under blue LEDs (Kim et al., 2014). Specifically, eight essential amino acids (L-allo-isoleucine, L-histidine, N6-acetyl-L-lysine, L-tryptophan, γ-glutamyl-L-valine, L-γ-glutamyl-L-leucine, γ-glutamylmethionine and γ-glutamylphenylalanine) increased in abundance under red light (**Figure 5A**) and could be leveraged on to improve availability and accessibility of these essential amino acids for human health (Sá et al., 2020).

Transcriptome analysis has been used to elucidate the annotation of different gene functions and biosynthetic pathways (Zhong et al., 2021). Sweet potato, as a vegetable, is a source of essential nutritional compounds beneficial to human health, including carotenes, chlorophylls, vitamins, flavonoids and phenolics (Li et al., 2020). In this study, the transcript profiles revealed that genes involved in anthocyanin and carotenoid biosynthesis were more highly expressed under blue light than under red light conditions (**Figure S3**). These compounds work as antioxidants beneficial to human health (Li et al., 2020). Anthocyanins are phenolic (flavonoid) compounds. They are used as natural-coloring agents found in diverse plants as dietary antioxidants, and their consumption could help boost health by supplying several nutrients (Yousuf et al., 2016). Also, studies have shown that anthocyanins possess antidiabetic, anticancer, anti-inflammatory, antimicrobial and anti-obesity effects, with the ability to prevent cardiovascular diseases (He et al., 2011). It has been shown that blue and UV-A light significantly increase the content of carotenoids and anthocyanins in Chinese kale and pak choi leaves (Li et al., 2020). Ouyang et al. (2015) reported that plant growth and development are regulated by light through altered gene expression. Also, the risk of developing cancer, cardiovascular disease, age-related macular degeneration, cataracts, disorders linked with inadequate immune function, and other degenerative diseases may be decreased by eating foods rich in carotenoids (Perera and Yen, 2007; Gammone et al., 2015). Genes identified in this study may be targeted for functional validation and verification to discover the regulatory role that they play in enhancing the nutritional profile of sweet potato leaves.

Our findings further explained the differences in the expression of light-regulated genes in sweet potato leaves in response to red and blue LEDs. Additionally, Chen et al. (2021) noted that the strength or weakness in synthesizing important metabolites is always indicated by the up- or downregulation of genes in certain metabolic pathways. Moreover, the development and metabolic changes in green plants are primarily thought to be regulated by changes in the expression of light-regulated genes (Ouyang et al., 2015). It has been reported that 16 hours of supplementation with blue lights increased the contents of carotenoids and anthocyanin, thereby increasing the antioxidant capacity of lettuce seedlings (Li et al., 2020). Earlier studies have shown that light significantly induces the production of phenolics, flavonoids, carotenoids and anthocyanin in several plants. For instance, UV-B, UV-A and blue light were cited to trigger the gene expression of some key enzyme functions in the biosynthesis of these metabolites (Li et al., 2020). Our results demonstrate a similar trend, where blue lights were shown to increase the expression of genes encoding anthocyanin and chlorophyll biosynthetic pathways. However, studies have shown that light quality significantly affects the accumulation of metabolites in crops grown in a controlled environment. Furthermore, the proportion of blue light supplied by LEDs affects the profiles of phytochemicals, depending on the species and the plant development stage of crops (Qinglu, 2021).

## 5 Conclusion

Sweet potato is a staple food and a critical food security crop in developing countries. However, previous studies have mainly focused on its production efficiency and tuber yield. The present study evaluates the influence of red and blue LEDs on sweet potato leaves. Blue light boosted dry matter accumulation, protein, total phenolics, flavonoids, and antioxidant scavenging activity relative to cultivation under red LEDs. Metabolite profiling using UPLC−MS/MS uncovered an abundance of 95 significant metabolites, where 77 compounds accumulated at higher levels in the leaf samples grown under red light and 18 metabolites at higher levels in leaves grown under blue light. KEGG pathway enrichment analysis revealed that nine and five DAMs were enriched in linoleic acid biosynthesis and alpha-linolenic acid metabolism.

**Figure 8:**
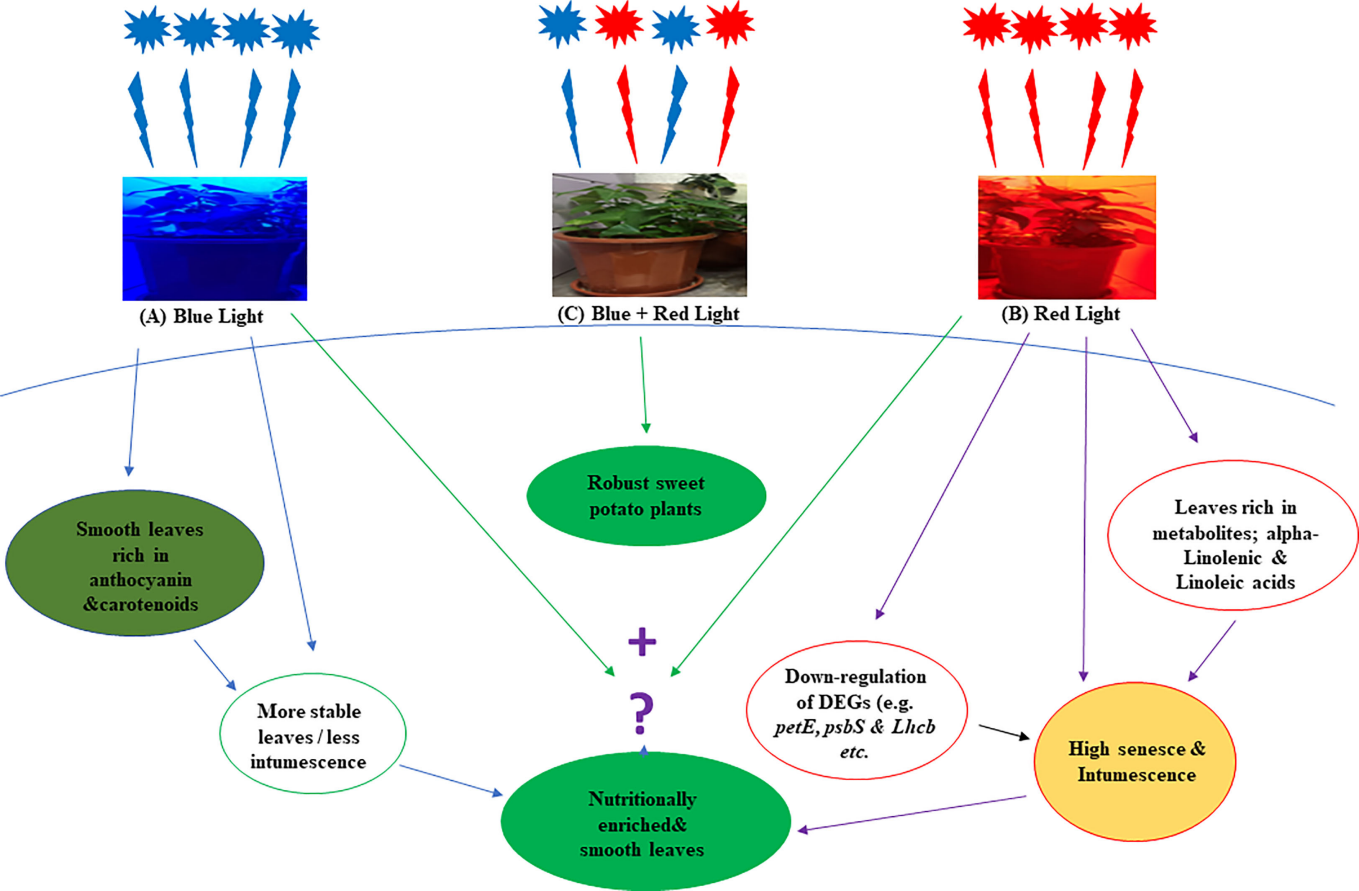
A hypothetical overview of the effect of red and blue LEDs on the production of sweet potato (I. batatas (L.) Lam) leaves. The leaves grown with monochromatic blue light were more stable **(A)** They have less intumescence and are rich in anthocyanin and carotenoids than those grown with monochromatic red lights, which have more abscission and intumescence **(B)** More DEGs were down-regulated under red light, and the leaves exhibit high metabolites (alpha-Linolenic and Linoleic acids) content. Hence, we proposed that a right proportion of red and blue light **(C)** may produce smooth leaves rich in metabolites.

Moreover, RNA sequencing identified 17,995 genes, out of which 14,039 novel genes were annotated. Most of the genes were upregulated in leaves grown under blue LEDs. The downregulation of the photosynthetic genes may account for the abscission and edema in leaves grown under red LEDs, and potentially serve as a putative mechanism of red and blue LED regulation of metabolites in sweet potato leaves was summarized in **Figure 8**. The results could guide breeders and agronomists in improving the quality of sweet potato leaves through breeding and improved production practices.

## Data availability statement

The datasets presented in this study can be found in online repositories. The names of the repository/repositories and accession number(s) can be found below: (http://www.ncbi.nlm.nih.gov/bioproject/947576), PRJNA947576.

## Author contributions

SAT: Designed and executed the experiment, analyzed the data, and wrote the original manuscript. JKA and BK re-analyzed the data, revised and formatted the manuscript. CL, JD, JL, JW, HH, QF, LC, and PH, helped experimenting and data collection. XC and DQ conceptualization, supervised the work and finalized the manuscript. All authors contributed to the article and approved the submitted version.
