# Supplementary material for: Integrated metabolome and transcriptome analyses provide insight into the effect of red and blue LEDs on the quality of sweet potato leaves
Source: Front Plant Sci. 2023 May 30;14:1181680. doi: 10.3389/fpls.2023.1181680 (PMC10266350; doi:10.3389/fpls.2023.1181680)
Supplement: Supplementary file 1 [file DataSheet_1.docx]

**Integrated metabolome and transcriptome analyses provide insight into the effect of red and blue LEDs on the quality of sweet potato leaves**


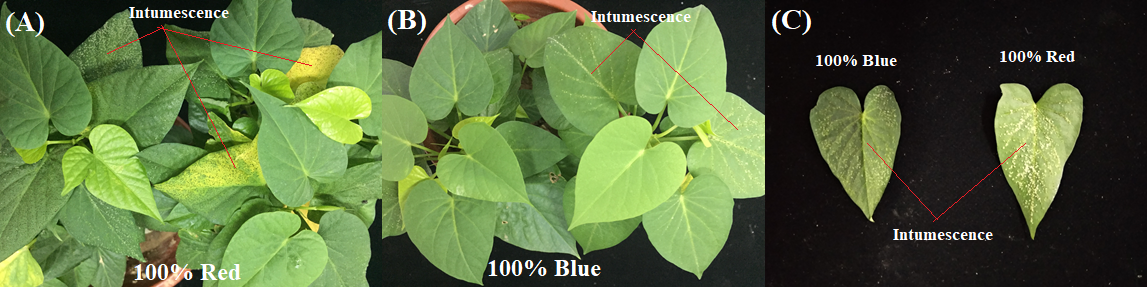


**Figure S1**: Sweet potato (*I. batatas* (L.) Lam) vines grown under red and blue LED lights. (**A**). vines exposed to red (100 %) and blue (100 %) LED lights. (**B**). vines showing the extent of leaf senescence (L2-red 100 %) and (L2-blue 100 %). (**C**). The intumescence is more apparent in the leaves grown under red LED light, and the leaves grown under blue look greener and intact.


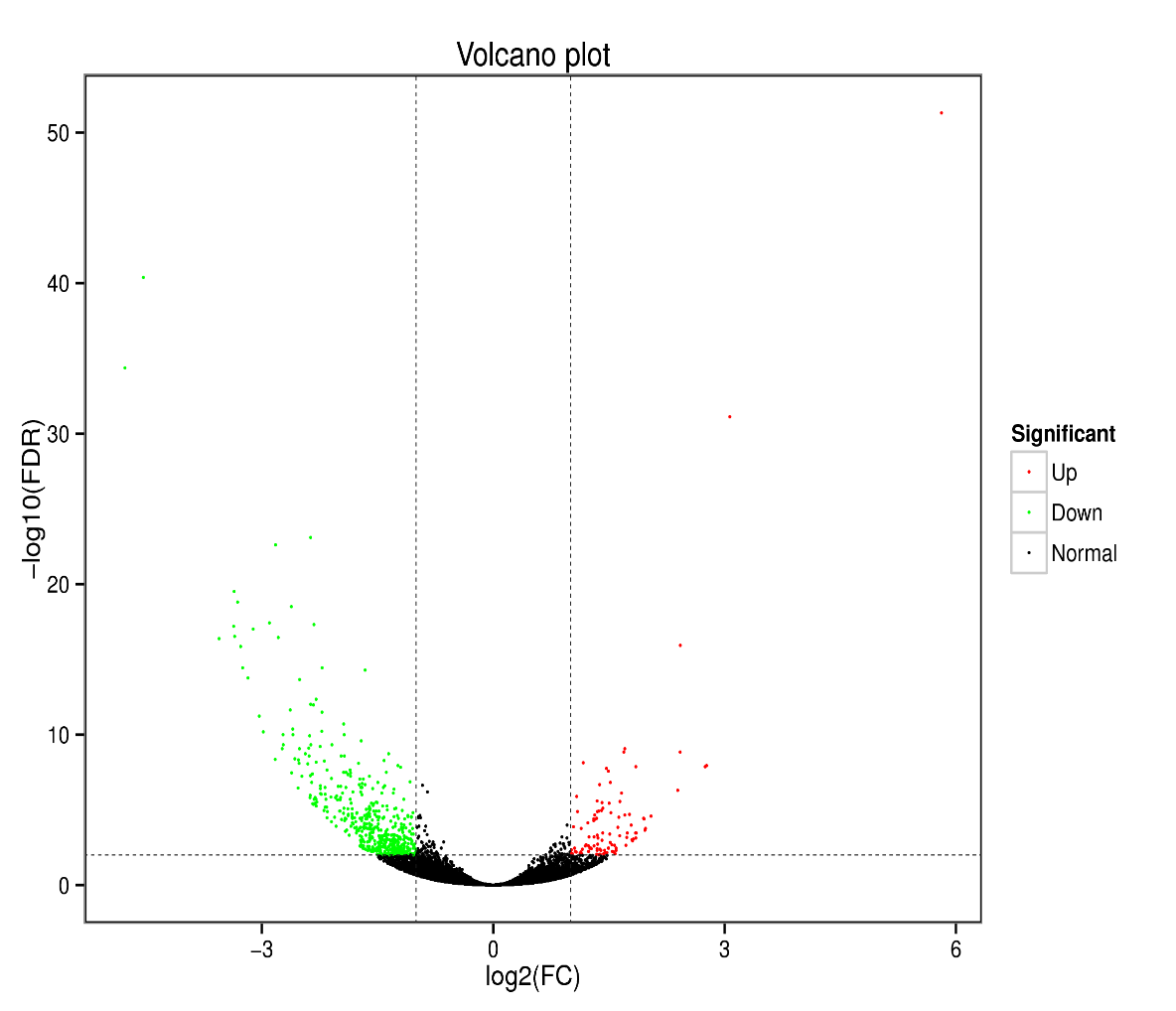


**Figure S2.** Volcano plot showing a total of 615 detected DEGs, 510 down-regulated (expressed higher in blue LED treated sweet potato than red LED) and 105 up-regulated (expressed higher in red LED treated sweet potato than blue LED) in sweet potato (*I. batatas* (L.) Lam) leaves grown with red and blue LEDs.


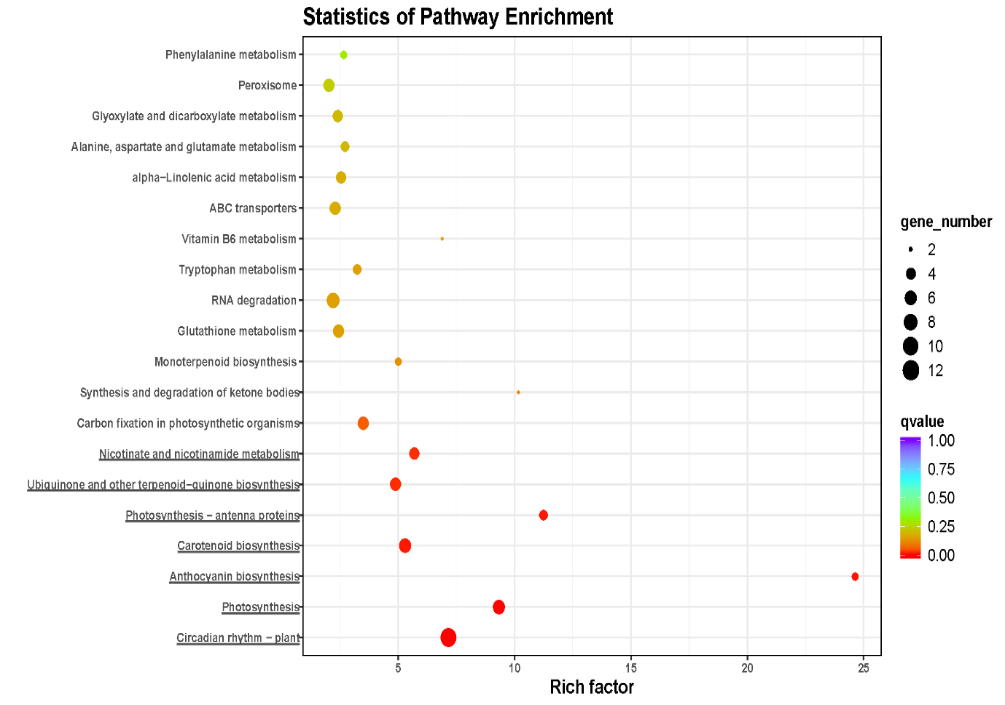


**Figure S3**: Top twenty Kyoto Encyclopedia of Genes and Genomes (KEGG) enriched pathway of sweet potato (*I. batatas* (L.) Lam) vines grown under red and blue LED. The underlined pathways on the left side of scatter plot were significantly differentiated between blue and red LED with a threshold of *P ≤ 0.05*.

**
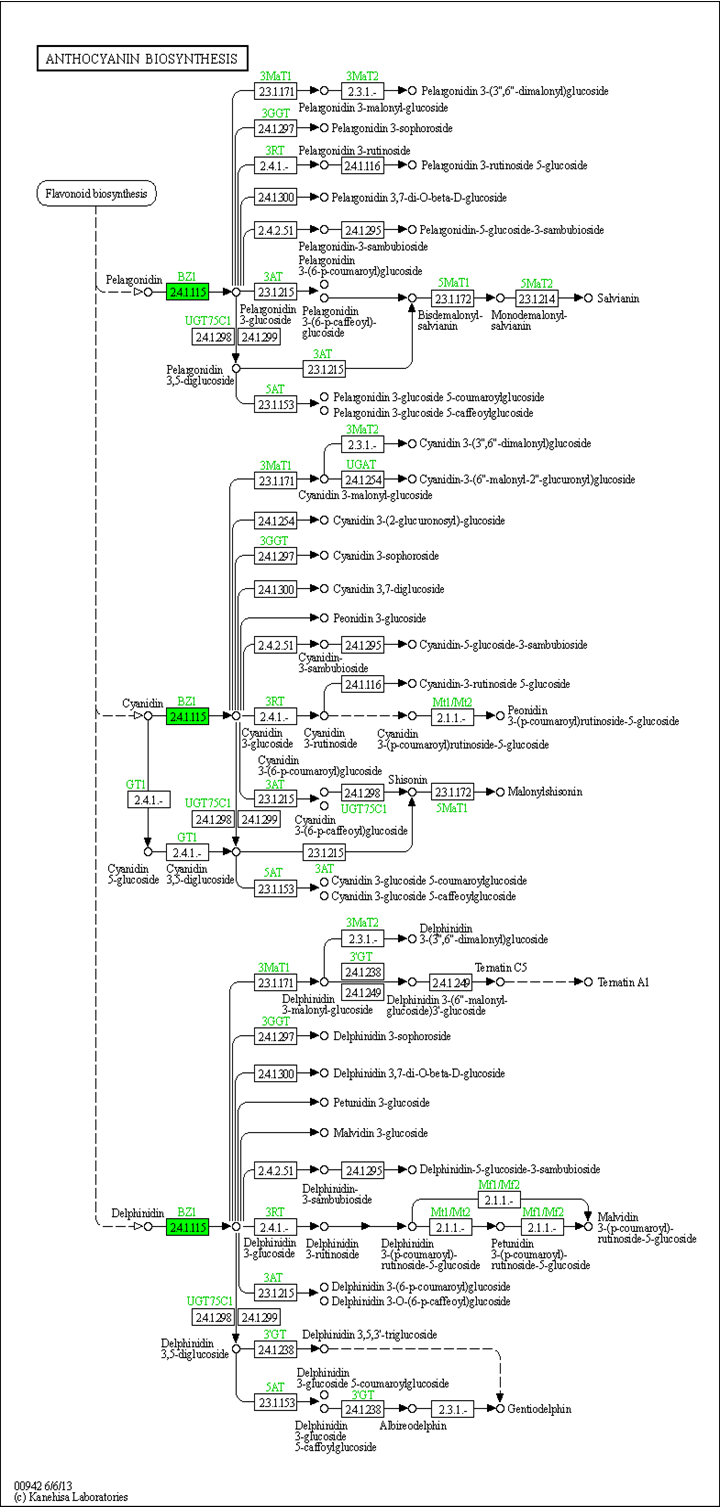
**

**Figure S4.** Anthocyanin biosynthetic pathway in comparison with blue and red blue LEDs used in growing sweet potato (*I. batatas* (L.) Lam) Three structural genes (*Ipomoea_batatas_newGene_14427*, *Ipomoea_batatas_newGene_29385* and *Ipomoea_batatas_newGene_7186*) encode for anthocyanidin 3-O-glucosyltransferase [EC:[24.1.115](https://www.genome.jp/entry/2.4.1.115)], designated BZI. See Figure 4A for their expression profile (log2 transformed of fragments kilobase of exon per million fragments mapped).


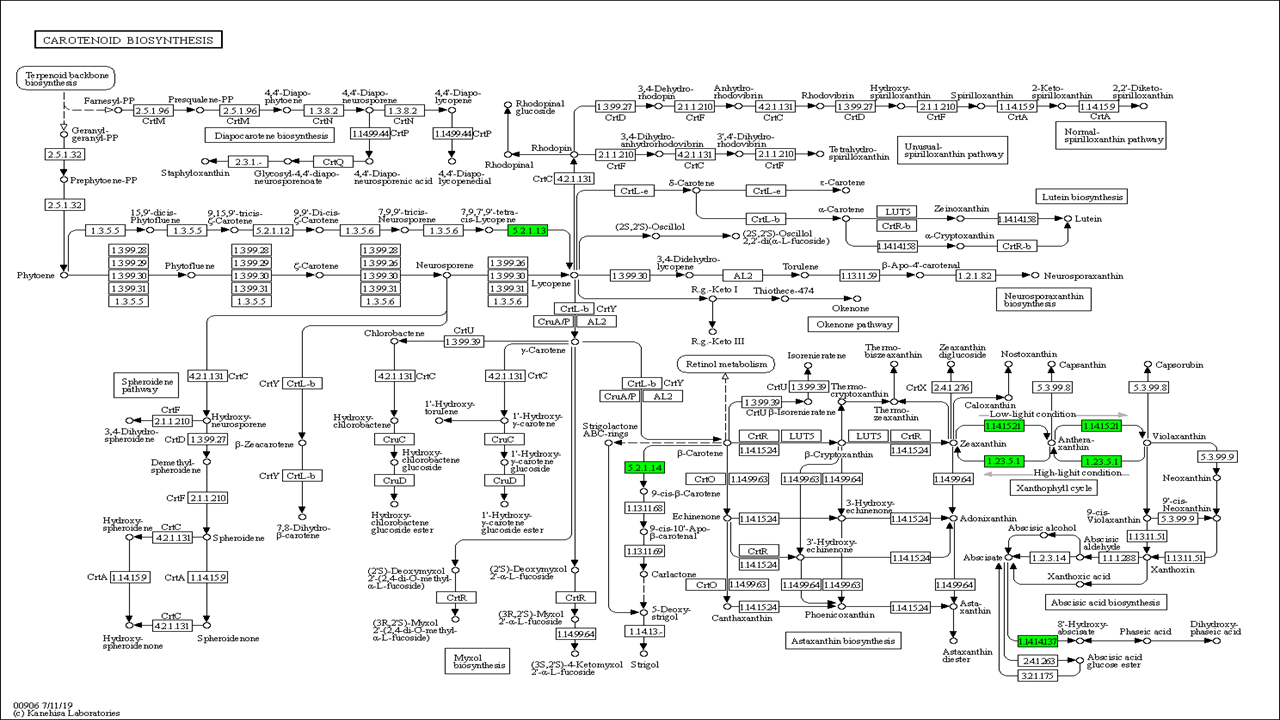


**Figure S5.** Carotenoid biosynthetic pathway in comparison with blue and red LEDs used in growing sweet potato (*I. batata* (L.) Lam). Seven structural genes, including two genes (*Ipomoea_batatas_newGene_14029* and *Tai6.24200*) encode carotenoid isomerase (crtH,crtISO) [EC:5.2.1.13], one gene (*Ipomoea_batatas_newGene_26163*) encode beta-carotene 9-cis-all-trans isomerase [EC:5.2.1.14], one gene (*Tai6.33677*) encode for zeaxanthin [EC:1.14.1521], one gene (*Tai6.4118*) antheraxanthin[EC:1.23.5.1] and two genes (*Ipomoea_batatas_newGene_7887* and *Tai6.39633*) encode for abscisate [EC:1.14.14137]. See Figure 4A for their expression profile (log2 transformed of fragments kilobase of exon per million fragments mapped).
